# Effects of exercise during chemotherapy for breast cancer on long-term cardiovascular toxicity

**DOI:** 10.1136/openhrt-2023-002464

**Published:** 2023-10-30

**Authors:** Willeke R Naaktgeboren, Martijn M Stuiver, Wim H van Harten, Neil K Aaronson, Jessica M Scott, Gabe Sonke, Elsken van der Wall, Miranda Velthuis, Tim Leiner, Arco J Teske, Anne M May, Wim G Groen

**Affiliations:** 1 Psychosocial Research and Epidemiology, Antoni van Leeuwenhoek Netherlands Cancer Institute, Amsterdam, The Netherlands; 2 Julius Center for Health Sciences and Primary Care, University Medical Center Utrecht, Utrecht University, Utrecht, Netherlands; 3 Center for Quality of Life, The Netherlands Cancer Institute - Antoni van Leeuwenhoek Hospital, Amsterdam, The Netherlands; 4 Faculty of Health, Amsterdam University of Applied Sciences, Amsterdam, The Netherlands; 5 Department of Health Technology and Services Research, University of Twente, Enschede, Netherlands; 6 Rijnstate Hospital, Arnhem, Netherlands; 7 Memorial Sloan Kettering Cancer Center, New York, New York, USA; 8 Weill Cornell Medical College, New York, New York, USA; 9 Medical Oncology, The Netherlands Cancer Institute - Antoni van Leeuwenhoek Hospital, Amsterdam, The Netherlands; 10 Division of Internal Medicine and Dermatology, University Medical Centre, Utrecht, The Netherlands; 11 Netherlands Comprehensive Cancer Organisation, Nijmegen, The Netherlands; 12 Department of Radiology, Mayo Clinic, Rochester, Minnesota, USA; 13 Department of Radiology, UMC Utrecht, Utrecht, Netherlands; 14 Cardiology, University Medical Centre, Utrecht, The Netherlands; 15 Department of Medicine for Older People, Amsterdam UMC, Vrije Universiteit Amsterdam, Amsterdam, Netherlands; 16 Aging & Later Life, Amsterdam Public Health Research Institute, Amsterdam, Netherlands; 17 Amsterdam Movement Sciences, Ageing & Vitality, Rehabilitation & Development, Amstermdam, Netherlands

**Keywords:** heart failure, magnetic resonance imaging, echocardiography

## Abstract

**Objective:**

Animal data suggest that exercise during chemotherapy is cardioprotective, but clinical evidence to support this is limited. This study evaluated the effect of exercise during chemotherapy for breast cancer on long-term cardiovascular toxicity.

**Methods:**

This is a follow-up study of two previously performed randomised trials in patients with breast cancer allocated to exercise during chemotherapy or non-exercise controls. Cardiac imaging parameters, including T1 mapping (native T1, extracellular volume fraction (ECV)), left ventricular ejection fraction (LVEF) and global longitudinal strain (GLS), cardiorespiratory fitness, and physical activity levels, were acquired 8.5 years post-treatment.

**Results:**

In total, 185 breast cancer survivors were included (mean age 58.9±7.8 years), of whom 99% and 18% were treated with anthracyclines and trastuzumab, respectively. ECV and Native T1 were 25.3%±2.5% and 1026±51 ms in the control group, and 24.6%±2.8% and 1007±44 ms in the exercise group, respectively. LVEF was borderline normal in both groups, with an LVEF<50% prevalence of 22.5% (n=40/178) in all participants. Compared with control, native T1 was statistically significantly lower in the exercise group (β=−20.16, 95% CI −35.35 to −4.97). We found no effect of exercise on ECV (β=−0.69, 95% CI −1.62 to 0.25), LVEF (β=−1.36, 95% CI −3.45 to 0.73) or GLS (β=0.31, 95% CI −0.76 to 1.37). Higher self-reported physical activity levels during chemotherapy were significantly associated with better native T1 and ECV.

**Conclusions:**

In long-term breast cancer survivors, exercise and being more physically active during chemotherapy were associated with better structural but not functional cardiac parameters. The high prevalence of cardiac dysfunction calls for additional research on cardioprotective measures, including alternative exercise regimens.

**Trial registration number:**

NTR7247.

**WHAT IS ALREADY KNOWN ON THIS TOPIC**

- Exercise during adjuvant chemotherapy effectively prevents anthracycline-related cardiotoxicity in animals. Clinical evidence is, however, limited.

**WHAT THIS STUDY ADDS**

- This study provides insight into the long-term effects of exercise on cardiovascular toxicity by describing a better structural cardiac phenotype in breast cancer survivors who exercised during chemotherapy compared with non-exercise controls.

**HOW THIS STUDY MIGHT AFFECT RESEARCH, PRACTICE OR POLICY**

- The results of this study call for more research on cardioprotective measures, including alternative exercise regimens, to offset the increased risk of cardiac dysfunction in breast cancer survivors.

## Introduction

In light of the growing number of breast cancer (BC) survivors,^1^ evaluating the long-term consequences of BC treatment is increasingly important. Cardiovascular disease (CVD) is among the most severe sequelae of BC treatment, given its association with increased morbidity and mortality.^2^

Exercise during chemotherapy is an established strategy to enhance or maintain cardiorespiratory fitness (CRF) in BC patients undergoing treatment.^3^ Also, there is emerging evidence that weekly exercise during anthracycline-based chemotherapy may prevent detrimental cardiac changes up to the first year after treatment.^4–8^ This is supported by a biological rationale, with rodent studies almost unanimously reporting cardioprotective effects of exercise interventions started before, during or after treatment with anthracyclines.^9^ However, if and to what extent this translates into better long-term cardiac measurements in BC survivors is unknown.

The current study evaluated the effects of an aerobic and resistance exercise intervention during adjuvant chemotherapy for BC on mitigating long-term cardiotoxicity and impaired CRF compared with non-exercise controls. Given that anthracycline-based chemotherapy is considered to impact CVD risk significantly, we hypothesised that if exercise is capable of yielding cardioprotection during this relatively short but intense period, allocation to an exercise intervention during chemotherapy will translate into reduced long-term cardiovascular toxicity. In addition, we hypothesised that BC survivors who are more active years after treatment have better CRF.

## Methods

### Study participants and design

**Figure 1:**
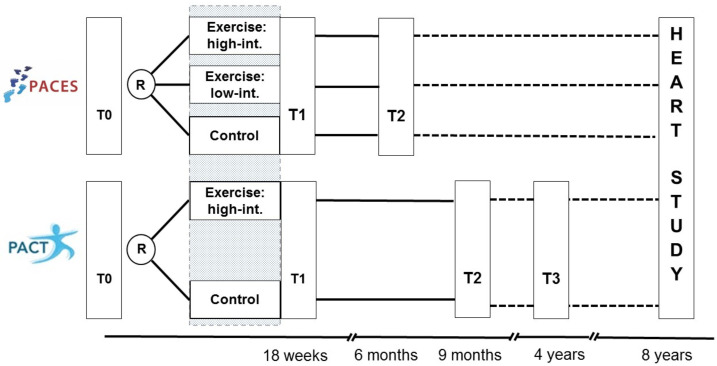
Original PACT and PACES study, and the follow-up Pact-Paces-Heart study. PACES, Physical Exercise during Adjuvant Chemotherapy Effectiveness Study; PACT, Physical Activity during Cancer Treatment.

This study (registered with ICTRP, NTR7247) is a long-term follow-up (FU) investigation of two previously performed prospective randomised controlled trials; the Physical Activity during Cancer Treatment (PACT, N=204) study and the Physical Exercise during Adjuvant Chemotherapy Effectiveness Study (PACES, N=230), both conducted between 2009 and 2013.^10 11^ The PACT and PACES study included participants with non-metastatic BC, free or serious comorbidities (eg, CVD) that would impede physical activity. These studies reported the positive effects of an exercise programme during adjuvant chemotherapy for non-metastasised BC on various outcomes, including fatigue, exercise capacity and symptom burden. In PACT, participants were randomised to either an 18-week, supervised moderate-to high-intensity aerobic and resistance exercise programme or usual care. PACES was a three-armed trial, where participants were randomly allocated to a supervised moderate-to high-intensity aerobic and resistance exercise programme (comparable to PACT’s intervention), a home-based low-intensity programme or usual care. The design and timing of data collection are shown in figure 1.

For the current study, all BC participants of the original PACT and PACES studies were, in principle, eligible. Exceptions were those who: (1) died during FU; (2) were not considered eligible by their treating physician (eg, too mentally burdensome or severe neuropathy); (3) received chemotherapy, targeted therapy or thoracic irradiation for recurrent or metastasised BC or other malignancies after the completion of the original PACT and PACES and (4) those who dropped out during the original PACT study (n=19) or actively declined invitations for future FU studies (n=11) at the time of the first FU study.^12^

### Study procedures

All PACT and PACES participants were screened for eligibility by their treating physician. If participants had died during FU, the cause of death (defined as cancer, CVD or other) was obtained. Eligible women were invited by their treating physician (or the study team for PACT participants who, during the first FU study, consented to be approached for future studies) for a one-time study visit at the UMCU. During the study visit, participants underwent a cardiac MRI including T1 mapping, echocardiography, a cardiopulmonary exercise test, a venous blood test and an interview (table 1; detailed information on study outcomes is provided in Supplementary file B). In addition, participants were asked to complete online questionnaires on patient-reported outcomes (data not included in the current manuscript).

**Table 1:** Outcomes measures of the Pact-Paces-Heart study

Measure instrument	Assessment	Parameter(s)
Cardiac MRI	Cardiac structure	T1 mapping: native T1 and ECV, and LGE
	Cardiac function	Left-ventricular EF, right-ventricular EF
Echocardiography	Cardiac deformation	Global longitudinal strain
	Diastolic function	Diastolic function
Cardiopulmonary exercise test	Cardiorespiratory fitness	VO_2_peak
Venous blood sample	Haematocrit, CV risk profile	Haematocrit, HbA1C, blood lipids, renal function.
SQUASH questionnaire	Self-reported physical activity	Moderate-to high-intensity leisure and sport physical activity, min/week
Face-to-face interview	Physical activity in the distant past	MET-hours/week

CV, cardiovascular; ECV, extracellular volume; EF, ejection fraction; LGE, late gadolinium enhancement; MET, metabolic equivalent task; SQUASH, Short Questionnaire to Assess Health-Enhancing Physical Activity.

### Statistical analyses

Intention-to-treat linear or logistic regression models were used with treatment allocation (control vs exercise (ie, the PACT and PACES moderate-to high-intensity exercise groups) as independent variables and cardiovascular toxicity parameters as dependent variables. For the logistic models, we used the following cut-offs; left ventricular ejection fraction (LVEF)<50%/≥50%,^13^ global longitudinal strain (GLS) >−18%/≤−18%,^14^ native T1≥1020 ms/<1020 and extracellular volume (ECV)≥28%/<28%. The latter two are based on institutional reference values (UMCU). These analyses were repeated with self-reported PA during chemotherapy as the main independent variable, regardless of treatment allocation. This was defined as the change in the sum score of self-reported PA between the end of chemotherapy (T1) and (T0), expressed as a Z-score, given that the two studies used different questionnaires.^10 11^ The potential non-linearity of this relationship was evaluated using restricted cubic splines models.

All models were adjusted for study (PACT vs PACES), age, thoracic radiotherapy, cumulative doxorubicin (equivalent) dosage and treatment with or without trastuzumab. Also, the influence of cardiovascular risk factors was explored by adding a composite score to the adjusted model. Cardiovascular risk factors were defined as follows: hypertension as having a blood pressure ≥140 mm Hg (systolic) and ≥90 mm Hg 90 (diastolic), being treated with antihypertensive medication; hypercholesterolaemia as having a to total cholesterol ≥6.5 mmol/L and LDL≥3.5 mmol/L or being treated with lipid-lowering drugs; diabetes mellitus as having a HbA1c>42 mmol/L, or being treated with glucose-lowering medication; obesity as having a BMI>30 kg/m^2^; and smoking as being a current smoker. The interaction between treatment allocation and doxorubicin (equivalent) dosage was tested by adding an interaction term to the fully adjusted model. In case of evidence of interaction (p<0.10), subsequent stratified analyses were provided for the following doxorubicin (equivalent) dosage strata; ≤210 mg/m^2^, 210–300 mg/m^2^ and ≥300 mg/m^2^).

As sensitivity analyses, we explored whether adding data from participants in the low-intensity PACES programme to the exercise group changed the outcomes of the intention-to-treat analyses. Due to low numbers in the low-intensity group, we did not perform separate analyses. Also, for those outcomes expressed as proportions (ie, LVEF, GLS and ECV), we reanalysed the data using beta-regression models instead of linear models, assuming that a beta-distribution might better fit the outcomes.^15^ For the analysis of CRF, analyses were repeated without excluding data of participants with submaximal tests (n=22). Last, we repeated the analyses using multiple imputations (n=50) by fully conditional specification for ECV and GLS since missing data on these parameters exceeded 10% (11.6% and 17.15%, respectively) (MICE package). All analyses were performed with R (V.4.0.3.) and Rstudio software (V.1.3.1093).

## Results

**Figure 2:**
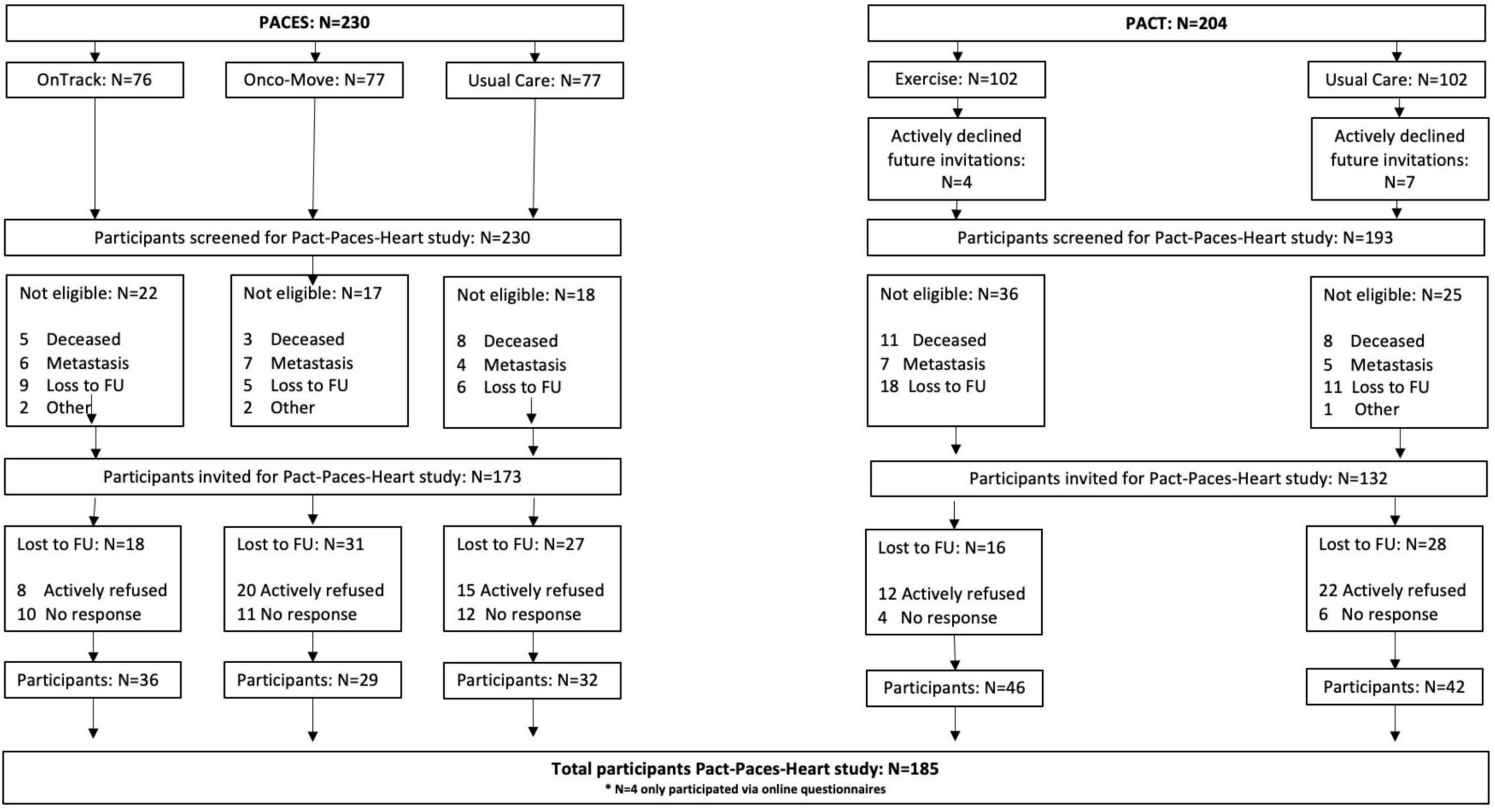
Flow chart of participants in the Pact-Paces-Heart study. FU, follow-up; PACES, Physical Exercise during Adjuvant Chemotherapy Effectiveness Study; PACT, Physical Activity during Cancer Treatment.

The mean interval between initial cancer diagnosis and completion of FU assessment was 8.5±1.1 years. Of the original PACT (N=204) and PACES (N=230) participants, 304 participants were invited for the FU study, of whom 185 women (n=185/304; 61%) were included. Ineligible participants were generally evenly distributed across study arms, and no cardiac-related deaths were recorded. Only four participants completed the online questionnaires. Thus, 181 patients were included in the current analyses (figure 2).

Participants were comparable to non-participants for most of the original PACT and PACES baseline characteristics (online supplemental table 1A,B), except that participants had a lower BMI, higher CRF before chemotherapy and a higher exercise intervention attendance rate.

10.1136/openhrt-2023-002464.supp1 Supplementary data

Of the 181 participants, 80 had been allocated to a moderate-to high-intensity exercise programme, 29 to the low-intensity exercise programme and 72 received care as usual. These groups had comparable characteristics during the FU study visit (table 2). All participants, except one, were treated with anthracyclines. The low-intensity exercise group had higher cumulative dosages (292 (240–332) mg/m^2^) and more often received trastuzumab (20.7%) compared with the moderate-to high-intensity and control group (235 (210–300) mg/m^2^ and 240 (210–300) mg/m^2^, and 16.7% and 11.3%, respectively). Cardiac comorbidities were documented for 23 participants, with a history of ischaemic heart disease being the most common diagnosis.

**Table 2:** Characteristics of the Pact-Paces-Heart study per study group at the time of study visit

	Control	Moderate-to high-intensity ET	Low-intensity ET
	**N=72**	**N=80**	**N=29**
Age, years	58.5±7.5	59.1±7.2	59.5±10.2
Original study			
PACT, %	40 (55.6)	46 (57.5)	0 (0)
PACES, %	32 (44.4)	34 (42.5)	29 (100)
Follow-up time, years	8.6±1.1	8.4±1.2	8.9±0.9
Menopausal status, %			
Premenopausal	4 (5.6)	6 (7.5)	2 (6.9)
Postmenopausal	67 (93.0)	73 (91.3)	27 (93.1)
Unknown	1 (1.4)	1 (1.2)	0 (0.0)
Receptor status			
Triple-negative	9 (12.5)	16 (20.0)	4 (13.8)
ER/PR+, HER2+	10 (13.9)	13 (16.2)	6 (20.7)
ER/PR-, HER2+	3 (4.2)	8 (10.0)	1 (3.4)
ER/PR+, HER2-	50 (69.4)	43 (53.8)	18 (62.1)
Radiotherapy (RT), %			
No RT	18 (25.0)	20 (25.0)	9 (31.0)
Left-sided	27 (37.5)	33 (41.2)	12 (41.4)
Right-sided	27 (37.5)	27 (33.8)	8 (27.6)
Anthracyclines, %*			
No anthracyclines	0 (0)	1 (1.3)	0 (0)
Doxorubicin	41 (56.9)	39 (48.8)	22 (75.9)
Epirubicin	30 (41.7)	39 (48.8)	6 (20.7)
Unknown	2 (2.8)	0 (0)	1 (3.4)
Cumul. dox. (equi.) dose, mg/m†	240 (210–300)	237 (210–300)	293 (241–352)
Trastuzumab, %	8 (11.1)	13 (16.2)	6 (20.7)
Medication use, %			
Cardiovascular	12 (16.7)	18 (22.5)	6 (20.7)
Anti-diabetic	3 (4.2)	1 (1.2)	1 (3.4)
Statins	6 (8.3)	5 (6.2)	3 (10.3)
Hormonal replacement	16 (22.2)	8 (10.0)	4 (13.8)
Others	31 (43.1)	23 (28.7)	4 (13.8)
Any comorbidity, %	31 (43.1)	28 (35.0)	8 (27.6)
Cardiovascular risk factors, %‡			
Hypertension	17 (23.6)	25 (31.2)	7 (24.1)
Hypercholesterolaemia	19 (26.4)	34 (42.5)	8 (27.6)
Diabetes mellitus	8 (11.1)	2 (2.5)	2 (6.9)
Obesity	10 (13.9)	14 (17.7)	2 (6.9)
Smoking, current	3 (4.2)	2 (2.5)	1 (3.4)
Cardiac comorbidities, %§			
Arrhythmias	5 (6.9)	3 (3.8)	0 (0.0)
Ischaemic heart disease	2 (2.8)	2 (2.5)	3 (10.3)
Impaired EF/heart failure	1 (1.4)	4 (5.0)	0 (0.0)
Other	1 (1.4)	1 (1.2)	0 (0.0)
None	63 (87.5)	70 (87.4)	26 (89.7)
Physical activity before diagnosis¶			
PACT, min/week	180.0 (60–365)	180.0 (110–270)	NA
PACES, sum score	64 (41–107)	80 (54–121)	79 (46–148)
Unknown	2 (2.8)	1 (1.2)	1 (3.4)

Presented as mean±SD, median (IQR) or number (percentages).

*Some patients have received both doxorubicin and epirubicin.

†Calculated using Doxorubicin: Epirubicin ratio=1:0.7.

‡Cardiovascular risk factors are defined as follows: hypertension=having a blood pressure higher than 140 mm Hg (systolic) and 90 mm Hg (diastolic) or being treated with antihypertensive medication. Hypercholesterolaemia=having total cholesterol ≥6.5 mmol/L and LDL≥3.5 mmol/L or being treated with lipid-lowering drugs. Diabetes mellitus=HbA1 c >42 mmol/L, or being treated with glucose-lowering medication. Obesity=having a BMI>30 kg/m^2^ Smoking=current smoker.

§Only those requiring treatment. Four participants (in the control arm) already had documented cardiac comorbidities (arrhythmias) at baseline.

¶The original PACT and PACES study used different questionnaires to assess physical activity before diagnosis; the SQUASH and PASE, respectively. The SQUASH score indicates minutes per week of moderate-to high-intensity leisure and sports physical activity, defined as any activity corresponding with an metabolic equivalent task value of 4 and higher. The PASE score combines information on occupation, leisure and household activities and ranges from 0 to 793, where higher scores correspond with greater physical activity.

AC, anthracyclines; BMI, body mass index; cumul., cumulative; dox, doxorubicin; EF, ejection fraction; equi, equivalent; ET, exercise training; NA, not available; PACES, Physical Exercise during Adjuvant Chemotherapy Effectiveness Study; PACT, Physical Activity during Cancer Treatment; SQUASH, Short Questionnaire to Assess Health-Enhancing Physical Activity.

### Cardiovascular outcomes

Cardiac imaging parameters and CRF are shown in table 3.

**Table 3:** Cardiac outcomes per study group

Imaging modality	Parameter	Control	Mod.-to high-int. ET	Low-int. ET
		n=72	n=80	n=29
Cardiac MRI*				
	Extracellular volume fraction, %†	25.3±2.5	24.6±2.8	25.5±2.7
	ECV>28%‡	9 (14.3)	8 (11.4)	6 (22.2)
	Native T1, ms§	1026±51	1007±44	1029±51
	Native T1>1020 ms‡	35 (50.0)	29 (37.7)	15 (53.6)
	Late gadolinium enhancement, %¶			
	Mid-wall	14 (21.5)	21 (27.3)	5 (17.9)
	Sub-endocardial	0 (0.0)	1 (1.3)	0 (0.0)
	Other	0 (0.0)	1 (1.3)	2 (7.2)
	None	51 (78.5)	54 (70.1)	21 (75.0)
	Left ventricular EF, %	54.6±4.9	53.0±7.8	54.2±7.3
	LV EF<50%	12 (17.1)	22 (27.8)	6 (20.7)
	Left ventricular EDV, ml/m^2^	80.9±11.6	82.7±16.9	85.6±15.7
	Left ventricular ESV, ml/m^2^	37.6±8.9	39.8±13.9	39.6±12.6
	Right ventricular EF, %	57.2±4.8	55.1±6.6	57.4±5.3
	RV EF<50%	5 (7.1)	13 (16.5)	2 (6.9)
Echocardiography				
	Global long. strain, %**	−18.7±3.0	−18.3±2.9	−17.6±2.4
	GLS>-18%	23 (36.5)	26 (41.9)	15 (60.0)
	Diastolic function			
	Normal	34 (47.2)	38 (47.5)	13 (44.8)
	Intermediate	33 (45.8)	27 (33.8)	11 (37.9)
	Impaired	5 (6.9)	15 (18.8)	5 (17.2)
Cardiopulmonary exercise test††				
	VO_2_peak, ml/kg/min	25.5±5.8	24.9±6.5	24.0±7.1

Presented as mean±SD, median (IQR) or number (percentages).

*All MRI information in n=3 (1.7%) for the following reasons: non-MR compatible cardiac device (n=1) and severe claustrophobia (n=2).

†Missing in n=21 (11.6%) for the following reasons: no cardiac MRI performed (n=3), not possible to administrate IV contrast (n=8), technical issue (n=6), poor quality (n=3), no ECG gating possible due to atrial fibrillation (n=1).

‡Institutional reference values (UMCU).

§Missing in n=6 (3.3%) for the following reasons: no cardiac MRI performed (n=3), poor quality (n=2), no ECG gating possible due to atrial fibrillation (n=1).

¶Missing in n=11 (6.7%) for the following reasons: no cardiac MRI performed (n=3), not possible to administrate intravenous contrast (n=8).

**Missing in n=31 (17.1%) for the following reason: imaging quality too poor for strain analysis (n=30) and atrial fibrillation (n=1).

††Missing in n=30 (16.6%) for the following reasons: submaximal exercise test (n=22), logistic constraints (n=3), newly-diagnosed atrial fibrillation (n=1), severe hypertension (n=1), newly-diagnosed severe left-ventricular dysfunction on cardiac imaging (n=1), unwillingness to perform the test (n=1), hamstring injury (n=1).

ECV, extracellular volume; EF, ejection fraction; ET, exercise training; GLS, global longitudinal strain; LV, left ventricular; RV, right ventricular; UMCU, University Medical Centre Utrecht.

For ECV, mean values were within normal ranges for most participants; 25.3%±2.5% in the control group, 24.6%±2.8% in the moderate-to high-intensity exercise group, and 25.5%±2.7% in the low-intensity exercise group. The proportion of survivors with abnormal values was 14.3% (N=9/63), 11.4% (N=8/70) and 22.2% (N=6/27), respectively. Mean native T1 was normal in the moderate-to high-intensity exercise group (1007±44 ms) but elevated in the control group (1026±51 ms) and the low-intensity exercise group (1029±51 ms).

**Figure 3:**
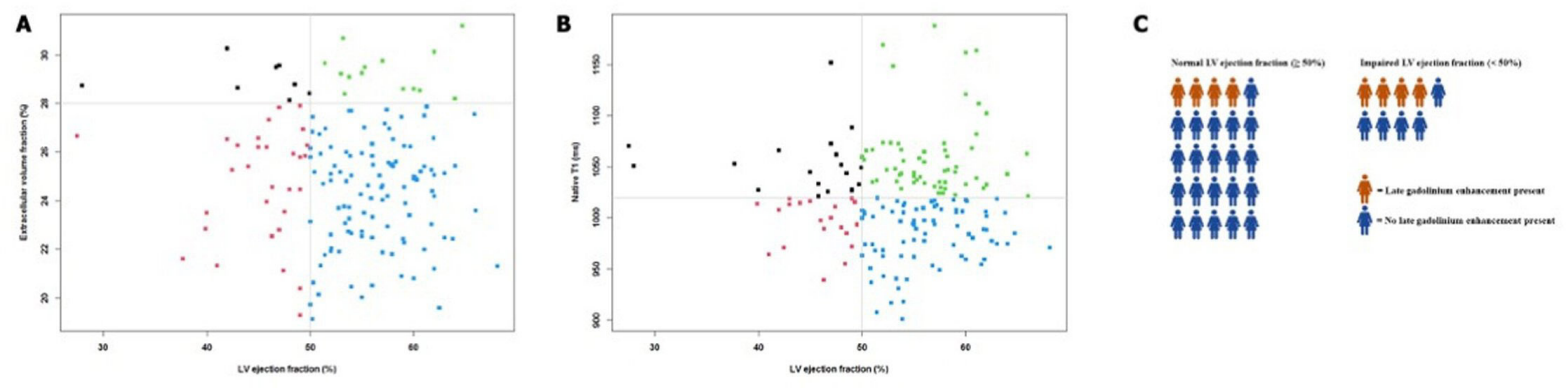
Relationship between structural and functional cardiac parameters using cardiac MRI. In (A, B), the relationship between ECV and native T1 and LVEF is depicted, respectively. The grey lines indicate the thresholds used for cut-off. In (C), the presence and absence of LGE in patients with normal and impaired LVEF is described. Here, each woman symbol represents five participants. ECV, extracellular volume; LGE, late gadolinium enhancement; LVEF, left ventricular ejection fraction.

Abnormal native T1 values were prevalent at 37.7% (N=29/77), 50% (N=35/70) and 53.6% (N=15/28), respectively. Late gadolinium enhancement (LGE) was present in approximately 25% of participants, almost all with mid-wall enhancement. We found borderline normal mean LVEF values in all three groups, with the lowest mean LVEF in the moderate-to high-intensity exercise group (53.0%±7.8%). This group also had the highest proportion with an LVEF<50%: 27.8% (N=22/79), followed by 20.7% (N=6/29) in the low-intensity exercise group and 17.1% (N=12/70) in the control group. The prevalence of abnormal ECV and native T1 was similar between those with impaired and normal LVEF, although LGE was more common in those with impaired LVEF (figure 3). For GLS, mean values were borderline normal in all groups. However, abnormal values were common. Diastolic function was normal in nearly half of the participants, with most diastolic dysfunction reported in the moderate-to high-intensity exercise group (N=15/80, 18.8%). CRF at FU was comparable across study arms.

### Effect of exercise during chemotherapy on long-term cardiovascular toxicity

We did not find a significant effect of moderate-to high-intensity exercise programme on ECV (β=−0.69, 95% CI: −1.62 to 0.25) nor on abnormal ECV (OR=0.76, 95% CI 0.24 to 2.34, table 4). Native T1 was statistically significantly lower in the exercise group compared with controls (β=−20.16, 95% CI −35.35 to −4.97). The odds of having an abnormal native T1 appeared lower in the exercise group (OR 0.53, 95% CI 0.26 to 1.07). We found no benefit of exercise for LVEF or GLS (β=−1.36, 95% CI −3.45 to 0.73 and β=0.31, 95% CI −0.76 to 1.37), nor on the likelihood of having an abnormal LVEF or GLS (OR 1.67, 95% CI 0.72 to 3.99); OR 1.34, 95% CI 0.63 to 2.88), respectively. Also, we found no significant effect of exercise on VO_2peak_ (β=0.21, 95% CI:−1.69 to 2.10). Additional correction for the presence of CV risk factors yielded comparable results. We found an interaction between doxorubicin equivalent dosage and exercise only for ECV (p=0.05). Subsequent stratified analyses did not generate a clear dose–response relationship, although the largest effect was observed among those treated with higher dosages (first stratum: β=−0.56, 95% CI −2.09 to 0.98), second stratum: β=−0.42, 95% CI −2.41 to 1.57 and third stratum: β=−0.76, 95% CI −2.15 to 0.64). The sensitivity analyses, where data of the participants in the low-intensity were combined with those in the moderate-to high-intensity exercise group and with multiple imputation for ECV and GLS, did not change the overall conclusions (online supplemental tables 2 and 3, respectively). The sensitivity analysis with beta-regression models (for ECV, GLS and LVEF) and those without exclusion of submaximal CPETs yielded similar results to those generated with the former analyses (data not shown).

**Table 4:** Effect of participation in a moderate-to high-intensity exercise programme during chemotherapy on cardiac outcomes based on an intention-to-treat analysis

Imaging modality	Parameter	Regression model	Unadjusted estimate (95% CI)	Partially adjusted* estimate (95% CI)	Fully adjusted† estimate (95% CI)
Cardiac MRI					
	ECV	Linear	–0.79 (−1.69, 0.11)	–0.80 (−1.71, 0.11)	–0.69 (−1.62, 0.25)
	ECV (>28%)	Logistic	0.77 (0.27, 2.16)	0.71 (0.23, 2.13)	0.76 (0.24, 2.34)
	Native T1	Linear	**–19.89 (−35.12, –4.66**)	**–20.58 (−35.41, –5.75**)	**–20.16 (−35.35, –4.97**)
	Native T1 (>1020 ms)	Logistic	0.60 (0.31, 1.16)	0.56 (0.28, 1.10)	0.53 (0.26, 1.07)
	LVEF	Linear	–1.67 (−3.79, 0.45)	–1.51 (−3.61, 0.60)	–1.36 (−3.45, 0.73)
	LVEF (<50%)	Logistic	1.87 (0.86, 4.23)	1.85 (0.82, 4.34)	1.67 (0.72, 3.99)
Echocardiography					
	GLS	Linear	0.40 (−0.63, 1.42)	0.37 (−0.68, 1.42)	0.31 (−0.76, 1.37)
	GLS (>-18%)	Logistic	1.26 (0.61, 2.59)	1.30 (0.61, 2.77)	1.34 (0.63, 2.88)
Cardiopulmonary exercise testing					
	VO_2_peak	Linear	0.68 (−2.83, 1.47)	0.13 (−2.08, 1.82)	0.21 (−1.69, 2.10)

*Partially adjusted includes adjustments for age, radiotherapy (none vs left sided or right sided), cumulative doxorubicin equivalent dosage, trastuzumab treatment and study (PACT vs PACES).

†Fully adjusted is the partially adjusted model with extra adjustment for cardiovascular risk factors (hypertension, hypercholesterolaemia, diabetes mellitus, obesity and being a current smoker; none vs 1 or >1).

ECV, extracellular volume; GLS, global longitudinal strain; LVEF, left ventricular ejection fraction; PACES, Physical Exercise during Adjuvant Chemotherapy Effectiveness Study; PACT, Physical Activity during Cancer Treatment.

### Self-reported physical activity during chemotherapy and long-term cardiovascular toxicity

The associations between the change of PA during chemotherapy (ie, independent of original treatment allocation) and long-term cardiovascular parameters are presented in table 5. An increase in PA was associated with better ECV (β=−0.48, 95% CI −0.95 to −0.01) and native T1 values (β=−8.54, 95% CI −16.47 to −0.16). Higher PA levels were also non-statistically significantly associated with better other cardiac imaging parameters and CRF.

**Table 5:** Association between change in self-reported physical activity during chemotherapy and cardiac outcomes

Imaging modality	Parameter	Regression model	Unadjusted estimate (95% CI)	Partially adjusted* estimate (95% CI)	Fully adjusted† estimate (95% CI)
Cardiac MRI					
	ECV	Linear	–0.39 (−0.83, 0.05)	**–0.47 (−0.93 to to –0.01**)	–0.48 (−0.95 to to 0.01)
	ECV (>28%)	Logistic	0.73 (0.44, 1.20)	0.66 (0.37, 1.15)	0.66 (0.36, 1.18)
	Native T1	Linear	–6.92 (–14.72, 0.89)	**–8.78 (−16.44, to –1.13**)	**–8.54 (−16.47, to –0.61**)
	Native T1 (>1020 ms)	Logistic	0.84 (0.60, 1.15)	0.78 (0.55, 1.10)	0.77 (0.54, 1.10)
	LVEF	Linear	–0.25 (−1.27, 0.77)	–0.13 (−1.18, 0.92)	0.10 (−0.97, 1.16)
	LVEF (<50%)	Logistic	0.97 (0.67, 1.43)	0.97 (0.66, 1.44)	0.87 (0.57, 1.32)
Echocardiography					
	GLS	Linear	–0.04 (−0.52, 0.44)	–0.05 (−0.55, 0.45)	–0.07 (−0.59, 0.44)
	GLS (>-18%)	Logistic	0.85 (0.60, 1.19)	0.86 (0.60, 1.21)	0.88 (0.61, 1.26)
Cardiopulmonary exercise testing					
	VO_2_peak	Linear	0.44 (−0.66, 1.53)	0.10 (−0.90, 1.09)	0.08 (−0.94, 1.09)

*Partially adjusted includes adjustments for age, radiotherapy (none vs left sided or right sided), cumulative doxorubicin equivalent dosage, trastuzumab treatment and study (PACT vs PACES).

†Fully adjusted is the partially adjusted model with extra adjustment for cardiovascular risk factors (hypertension, hypercholesterolaemia, diabetes mellitus, obesity and being a current smoker; none vs 1 or >1).

ECV, extracellular volume; GLS, global longitudinal strain; LVEF, left ventricular ejection fraction; PACES, Physical Exercise during Adjuvant Chemotherapy Effectiveness Study; PACT, Physical Activity during Cancer Treatment.

No evidence for non-linearity was found (p>0.10) (table 5).

### Physical activity years after treatment and cardiovascular toxicity

Levels of moderate-to high-intensity leisure and sports PA at FU were comparable for the control and the moderate-to high-intensity exercise group (155 (60–360) min/week and 150 (60–368) min/week, respectively) and higher in the low-intensity exercise group (240 (30–360) min/week). The MET-hours/week participants engaged in over the last years were comparable across the study arms (online supplemental table 4). We did not observe a statistically significant association between PA patterns at FU and cardiac imaging parameters, but higher PA levels at FU were associated with higher VO_2peak_ values (β=0.70, 95% CI 0.42 to 0.98, online supplemental table 5).

## Discussion

This analysis of a large cohort of BC survivors indicates that exercise during chemotherapy improved native T1 values but not ECV or functional cardiac imaging parameters after a mean FU time of 8.5 years. Higher self-reported PA levels during chemotherapy, independent of the exercise programme, were associated with better ECV and native T1 values. At FU, higher PA levels were associated with better CRF but not with cardiac imaging parameters. We found structural and functional cardiac abnormalities in a relatively high proportion of the BC survivors in our study.

Many rodent studies support the hypothesis that exercise during anthracycline-based chemotherapy yields short-term cardioprotection via, among other pathways, reducing the intracardial accumulation of anthracyclines.^9^ Exercise also has salutary effects on cardiac function in non-cancer populations.^16^ However, clinical trials in patients with cancer have yielded mixed results.^4–8^ Our study provides the first long-term results on the effect of exercise on long-term cardiovascular toxicity. We observed an effect of exercise and higher levels of PA native T1 (and on ECV in the analyses with self-reported PA) but not on functional cardiac parameters. Native T1 and ECV are relatively new parameters and are proposed as non-invasive surrogate markers of cardiac tissue composition.^17^ Native T1 is a composite signal of all cardiac T1 values, including intracellular and extracellular components. Since the myocardial volume primarily consists of cardiomyocytes, these cells form the bulk of the native T1 signal. ECV, by definition, represents the proportion of ECV, and it is assumed that ECV changes are primarily driven by interstitial fibrosis.^17^ These parameters have received increasing attention based on the assumption that tissue damage precedes functional decline. Indeed, most^18–21^ but not all^22^ studies have described elevated native T1 and ECV values in the context of preserved LVEF in adult cancer survivors treated with anthracyclines. In our study, we observed that native T1 abnormalities were prevalent, while ECV values were within normal ranges for most participants, suggesting that other mechanisms than fibrosis play a role. High native T1 values are reported in the context of (chronic) cardiac inflammation,^17^ while systemic anti-inflammatory effects are reported following exercise.^23^ Whether this could explain the finding of lower native T1 in the exercise group is worthy of future research. Of clinical importance, both native T1 and ECV have been described as independent prognostic factors for all-cause mortality or heart failure hospitalisation.^24 25^ Native T1 was even superior to LVEF in predicting all-cause mortality and major adverse cardiocerebrovascular events.^25^ This suggests that exercise during chemotherapy could be useful to offset the increased CVD risk in BC survivors. However, we observed a non-significant yet opposite trend on the functional cardiac parameters (ie, less favourable LVEF and GLS in the exercise group). Although we do not believe that this indicates a harmful effect of exercise on cardiac functioning, more high-quality, randomised studies that are prospectively designed to analyse cardiac outcomes during and after chemotherapy are needed to investigate the cardioprotective effects of exercise more robustly.

There is a large body of evidence supporting the positive effects of exercise on enhancing CRF and PA in the time frame surrounding BC treatment.^3^ A recent randomised study described that a 24-week exercise intervention performed during chemotherapy was more effective in terms of less CRF decline postchemotherapy compared with participants that participated in the exercise intervention after the completion of chemotherapy.^26^ However, less is known about the lasting effects of exercise interventions during chemotherapy on CRF and PA years after treatment. A 2-year FU study found, comparable to our results, no (significant) effect of exercise on CRF or PA.^27^ This contrasts with two other studies, including the former PACT FU study, which reported higher PA levels in BC survivors who exercised during chemotherapy after a FU of 4 and 5 years.^12 28^ A recent study found that a 24-week exercise intervention, performed both during or after chemotherapy, was associated with less decrease in CRF compared with controls, with larger effects in the group that exercised during chemotherapy

Given that PA levels years after treatment were associated with better CRF, and considering the importance of CRF in terms of symptom burden and survival after cancer,^29^ our results provide additional evidence that BC survivors could benefit from efforts to maintain their PA levels during (long-term) survivorship.

### Strengths and limitations

Strengths of our study include the in-depth cardiac assessment, including a multiparametric cardiac MRI scan, in combination with CRF and PA measurements, in a large sample after an appropriate FU period of, on average, 8.5 years post-treatment. The first potential study limitation is the possibility of selective response. Inherent to our study design, we cannot rule out that participation was related to (not) having CVD. Also, we observed a lower BMI, higher CRF before chemotherapy and a higher attendance rate among participants, compared with non-participants. This could have resulted in a lower prevalence of cardiac abnormalities among our relatively fit and healthy subset of participants, which could have limited our ability to study an effect of exercise on CVD. However, given that participation rates were similar across the trial arms with no cardiac deaths reported during FU and that some of our primary outcome parameters (ie, ECV and GLS) are related to subclinical (asymptomatic) CVD, a selective response seems less likely. A second limitation is that no pretreatment cardiac measurements were available. Hence, we could not establish baseline comparability between groups for these measures nor correct for any relevant baseline differences in our analyses. Third, GLS was missing for 31 participants (17.1%). This occurred more often in patients with left-sided than right-sided BC (58.1.% vs 49.3%) and with higher BMI (28.2±4.4 vs 25.3±4.0 kg/m^2^). This might have underestimated the prevalence of GLS abnormalities but is unlikely to influence the analysis of exercise effects. Multiple imputation analyses support the latter. Last, participants, relative to non-participants, had higher adherence rates, which might limit the generalisability of our results to the whole BC population.

### Future directions

We found an LVEF<50% prevalence in almost a quarter of our study population after a mean FU of 8.5 years, which is higher than described in a former study among anthracycline-treated patients.^30^ This is surprising, given our, apart from having BC, healthy sample at diagnosis. The clinical significance of this finding warrants further (longitudinal) exploration. Also, preclinical studies have used exercise interventions with volumes and intensities that would not be feasible in patients with cancer undergoing treatment,^9^ and a recent meta-analysis observed only a positive effect of exercise on LVEF when limiting the analyses to studies with ≥36 exercise sessions.^31^ Thus, future studies considering different exercise interventions or exercise interventions combined with other approaches (pharmacological adjuncts, dietary restrictions) may be needed to offset long-term cardiotoxicity.

### Conclusion

A supervised moderate-to-high intensity aerobic and resistance exercise intervention during chemotherapy improved native T1, but not ECV or functional cardiac imaging parameters, in long-term BC survivors. Self-reported increase in PA during chemotherapy was associated with better T1 mapping indices. Higher PA levels were associated with better CRF years after treatment but not with cardiac imaging parameters. The high prevalence of cardiac abnormalities calls for more research on cardioprotective measures, including alternative exercise regimens or pharmacological adjuncts, to offset the increased risk of cardiac dysfunction in BC survivors undergoing chemotherapy.

## Data Availability

Data are available on reasonable request.
