# Supplementary material for: Effects of exercise during chemotherapy for breast cancer on long-term cardiovascular toxicity
Source: Open Heart. 2023 Oct 30;10(2):e002464. doi: 10.1136/openhrt-2023-002464 (PMC10619040; doi:10.1136/openhrt-2023-002464)
Supplement: Supplementary data [file openhrt-2023-002464supp001.pdf]

Supplemental file A: additional information on study outcomes.

Cardiac MRI

Scans were performed on a 1.5T clinical MRI-scanner (Ingenia, Software release 4.1-5.3, Philips Healthcare). All exams (except two) were reviewed by the same cardiovascular imaging expert (TL) blinded to original treatment allocation. T1 maps were acquired in three short-axis views (basal, mid-ventricular, and apical) using a modified Look-Locker inversion (MOLLI) recovery sequence. Extracellular volume (ECV) fraction was calculated as (1 - hematocrit (%) * $\Delta R1_{myocardium}$*$\Delta R1_{blood}$), where ΔR1 is defined as 1/T1. T1 maps were anonymously analyzed using dedicated postprocessing software (Medis Suite version 3.2.48.2, MapMaker ECV Application version 2.2.44) by a single researcher (WN). Intra-rater reliability coefficient in a subset of 15 scans was 0.94 (95%CI 0.86;0.98) for ECV and 0.88 (95%CI 0.73;0.95) for native T1. For native T1 and ECV, the mean score of the three short-axis slices was used in the final analyses.

All patients underwent steady-state free precession sequences in multiple orientations for assessment of wall motion and measurement of functional parameters for both LV and right ventricle (RV). Except for ejection fraction (EF), all values were indexed to body surface area. All patients underwent viability and phase-sensitive inversion recovery enhancement imaging after administration of double-dose (0.2 mmol/kg) gadolinium-based contrast agent gadobutrol (Gadovist, Bayer Healthcare). Segmental late gadolinium enhancement patterns were classified as follows: subendocardial, mid-wall, transmural or epicardial.

The following imaging parameters were used:

- Field of view: 320x320 mm
- Matrix: 256x256
- Flip angle: 60°
- Parallel imaging acceleration factor: 3
- Slice thickness: 8.0mm
- Echo time: 1.7ms
- Repetition time; 4.0ms

T1 mapping parameters:

- Field of view: 300x300mm

- Matrix: 256x256
- Flip angle: 35°
- Parallel imaging acceleration factor: 2
- Slice thickness: 8.0mm
- Echo time: 1.1ms
- Repetition time; 4.0ms

Echocardiography

Transthoracic 2D-echocardiograms were acquired using a GE Vivid E9 or E95 machine. In line with current guidelines for adult cancer survivors [1], measurements of LV function, dimensions, and diastology were performed using one cardiac cycle. Diastolic function was scored as *normal, indeterminate,* or *diastolic dysfunction,* according to the most recent guidelines [2]. Global longitudinal strain (GLS) was assessed via 2D-speckle tracking echocardiography in the apical four-, three – and two-chamber views using offline EchoPAC software (version 2.03, GE Vingmed, Horten, Norway). All echocardiography data were collected by the same researcher (WN), after receiving sufficient training in GLS analysis [3], and were reviewed by the same cardiologist (AT), blinded for original treatment allocation.

Cardiopulmonary exercise tests

Cardiorespiratory fitness was assessed with a cardiopulmonary exercise test with continuous breathing gas analysis. Cycling workload increased every minute by 10,15 or 20 W till exhaustion, on the basis of participants' symptoms or at the discretion of the supervising physician. Peak oxygen uptake (VO_2Peak) was defined as the average value for the last 30 seconds before exhaustion and expressed in ml/kg/min. Only maximum exercise tests, defined as those where the respiratory exchange rate ≥1.1, were included in the analysis.

Venous blood sample

A fasting venous blood sample was taken to determine hematocrit (for ECV calculation), renal function, and presence of diabetes mellitus and hypercholesterolemia.

Physical activity

The original PACT and PACES study used different questionnaires to assess physical activity before diagnosis; the Short Questionnaire to Assess Health enhancing physical activity (SQUASH) [4] and the Physical Activity Scale for Elderly (PASE) [5], respectively. In the FU study, the former

questionnaire was used. This questionnaire comprises questions on commuting activing, leisure-time and sports activities, household activities and work-related activities. We calculated the minutes per week of moderate-to high-intensity leisure and sport physical activity, which include all activities that correspond with a MET-value of ≥ 3.0. To obtain an estimate for physical activity participants engaged in over the past years (i.e. since completion of the original PACT and PACES studies), we used a structured, face-to-face interview [6]. All interviews were performed by the same researcher (WN). Total physical activity scores were derived in MET-hours per week [6].

Supplemental references

1. J. C. Plana *et al.*, “Expert consensus for multimodality imaging evaluation of adult patients during and after cancer therapy: a report from the American Society of Echocardiography and the European Association of Cardiovascular Imaging.,” *Eur Heart J Cardiovasc Imaging*, vol. 15, no. 10, pp. 1063–1093, Oct. 2014, doi: 10.1093/ehjci/jeu192.
2. S. F. Nagueh *et al.*, “Recommendations for the Evaluation of Left Ventricular Diastolic Function by Echocardiography: An Update from the American Society of Echocardiography and the European Association of Cardiovascular Imaging.,” *J Am Soc Echocardiogr*, vol. 29, no. 4, pp. 277–314, Apr. 2016, doi: 10.1016/j.echo.2016.01.011.
3. J. Chan *et al.*, “Left Ventricular Global Strain Analysis by Two-Dimensional Speckle-Tracking Echocardiography: The Learning Curve.,” *J Am Soc Echocardiogr*, vol. 30, no. 11, pp. 1081–1090, Nov. 2017, doi: 10.1016/j.echo.2017.06.010.
4. G. C. W. Wendel-Vos, A. J. Schuit, W. H. M. Saris, and D. Kromhout, “Reproducibility and relative validity of the short questionnaire to assess health-enhancing physical activity.,” *J Clin Epidemiol*, vol. 56, no. 12, pp. 1163–1169, Dec. 2003, doi: 10.1016/s0895-4356(03)00220-8.
5. R. A. Washburn, E. McAuley, J. Katula, S. L. Mihalko, and R. A. Boileau, “The physical activity scale for the elderly (PASE): evidence for validity.,” *J Clin Epidemiol*, vol. 52, no. 7, pp. 643–651, Jul. 1999, doi: 10.1016/s0895-4356(99)00049-9.
6. M. E. Schmidt, T. Slanger, J. Chang-Claude, J. Wahrendorf, and K. Steindorf, “Evaluation of a short retrospective questionnaire for physical activity in women.,” *Eur J Epidemiol*, vol. 21, no. 8, pp. 575–585, 2006, doi: 10.1007/s10654-006-9042-9.

Supplementary table 1a. Characteristics at baseline of original PACT participants, and those of participants and non-participants of the Pact-Paces-Heart study. Presented as mean ± SD, median (interquartile range] or number (percentages).

	**Total PACT N=204**	**Non-participants N=116**	**Participants N=88***
Age, years	49.6 ± 8.0	49.2 ± 8.8	50.1 ± 6.9
Study group, %			
Control	102 (50.0)	60 (51.7)	42 (47.7)
Intervention	102 (50.0)	56 (48.3)	46 (52.3)
Treatment characteristics			
AC dose, mg/m^2	256.5 ± 79.9	260.6 ± 87.8	251.1 ± 68.4
Herceptin, %	30 (14.8)	16 (13.9)	14 (15.9)
Radiotherapy, %	141 (69.1)	81 (69.8)	60 (68.2)
Cardiovascular risk factors			
Hypertension, %†	25 (12.3)	15 (12.9)	10 (11.4)
Body mass index, kg/m^2	26.2 ± 4.8	26.8 ± 5.1	25.3 ± 4.4
Obesity, %‡	35 (17.2)	23 (20.0)	12 (13.6)
Any comorbidity, %	26 (12.7)	15 (12.9)	11 (12.5)
Physical fitness			
VO_2 $peak_{baseline}$	23.8 ± 5.4	22.9 ± 5.1	25.1 ± 5.5
VO_2 $peak_{end\ chemo}$	21.1 ± 5.3	20.7 ± 5.4	21.6 ± 5.1
Attendance rate§			
N of classes, %	83.3 (69.4-90.9]	78.8 (59.7-87.2]	86.1 (77.8-91.2]
>80%, %	54 (52.9)	21 (37.5)	33 (71.7)

* Of whom n=2 only participated via online questionnaires.
† Hypertension is defined as systolic blood pressure >140 mm Hg and diastolic blood pressure >90 mm Hg, or documented hypertension.
‡ Obesity is defined as having a BMI>30 kg/m^2. § Only applicable for participants in the exercise arm.

Supplementary Table 1b. Characteristics at baseline of original PACES participants, and those of participants and non-participants of the Pact-Paces-Heart study. Presented as mean ± SD, median (interquartile range] or number (percentages).

	**Total PACES N=230**	**Non-participants N=133**	**Participants[*] N=97**
Age, years	50.7 ± 9.1	50.6 ± 9.7	50.7 ± 8.4
Study group, %			
Control	77 (33.5)	45 (34.1)	32 (32.7)
Onco-Move	77 (33.5)	47 (35.6)	30 (30.6)
On-Track	76 (33.0)	40 (30.3)	36 (36.7)
Treatment characteristics			
AC dose, mg/m^2	280.2 ± 65.5	283.5 ± 67.0	276.1 ± 63.61
trastuzumab %	55 (23.9)	29 (22.0)	26 (26.5)
Radiotherapy, %	180 (79.3)	103 (79.8)	77 (78.6)
Cardiovascular risk factors			
Hypertension, %[†]	36 (15.7)	20 (15.2)	16 (16.3)
Body mass index, kg/m^2	26.0 ± 4.5	26.4 ±4.9	25.5 ± 3.8
Obesity, %[‡]	37 (16.2)	26 (20.0)	11 (11.2)
Smoking (baseline), %	29 (12.6)	18 (13.6)	11 (11.2)
Any comorbidity, %	75 (32.6)	15 (12.9)	11 (12.5)
Cardiac comorbidity, %	7 (3.0)	6 (4.5)	1 (1.0)
Physical fitness			
$MSEC_{baseline}$	256.1 ± 48.5	251.1 ± 50.5	262.9 ± 44.9
$MSEC_{end\ chemo}$	218.8 ± 61.5	208.3 ± 66.0	232.4 ± 52.4
Attendance rate[§]			
N of classes, %	76.7 (60.7-85.2]	74.5 (56.9-83.6]	79.1 (65.2-86.3]
>80%, %	30 (39.5)	13 (32.5)	17 (47.2)

---

* Of whom n=2 only participated via online questionnaires.

† Hypertension is defined as having a documented diagnosis of hypertension.

‡ Obesity is defined as having a BMI>30 kg/m^2.

§ Only applicable for participants in the supervised exercise arm.

Supplementary Table 2. Effect of randomization to an exercise program (low-intensity or a moderate-to high-intensity) during chemotherapy on cardiac outcomes based on an intention-to-treat analysis.

Imaging modality	Parameter	Regression model	Unadjusted estimate (95%CI)	Partially adjusted* estimate (95%CI)	Fully adjusted† estimate (95%CI)
Cardiac MRI					
	ECV	Linear	-0.52 (-1.36, 0.32)	-0.57 (-1.44, 0.30)	-0.47 (-1.33, 0.40)
	ECV (>28%)	logistic	1.01 (0.41, 2.58)	0.95 (0.37, 2.51)	0.98 (0.38, 2.64)
	Native T1	Linear	**-13.94 (-28.51, 0.63)**	**-19.03 (-33.32, -4.74)**	**-19.05 (-33.61, -4.48)**
	Native T1 (>1020 ms)	Logistic	0.72 (0.39, 1.32)	0.60 (0.31, 1.15)	0.57 (0.29, 1.10)
	LVEF	Linear	-1.31 (-3.33, 0.70)	-1.25 (-3.32, 0.82)	-1.01 (-3.06, 1.04)
	LVEF (<50%)	logistic	1.69 (0.81, 3.71)	1.70 (0.78, 3.88)	1.53 (0.69, 3.54)
Echocardiography					
	GLS	Linear	0.59 (-0.33, 1.52)	0.49 (-0.48, 1.46)	0.44 (-0.53, 1.41)
	GLS (>-18%)	Logistic	1.55 (0.80, 3.03)	1.48 (0.75, 2.98)	1.50 (0.76, 3.02)
Cardiopulmonary exercise testing					
	VO_2peak	Linear	-0.92 (-2.97, 1.12)	-0.34 (-2.27, 1.59)	-0.07 (-1.92, 1.79)

* Partially adjusted includes adjustments for age, radiotherapy (none versus left-sided or right-sided), cumulative doxorubicin equivalent dosage, trastuzumab treatment and study (PACT vs PACES).

† Fully adjusted is the partially adjusted model with extra adjustment for the presence of cardiovascular risk factors (hypertension, hypercholesterolemia, diabetes mellitus, obesity and being a current smoker; none versus 1 or >1).

Supplementary Table 3. Effect of participation in a moderate-to high-intensity exercise program during chemotherapy on cardiac outcomes based on an intention-to-treat analysis using unimputed versus imputed dataset.

Imaging modality	Parameter	Regression model	Unimputed analyses[*,†] estimate (95%CI)	Imputed analyses[*] estimate (95%CI)
Cardiac MRI				
	ECV	Linear	-0.69 (-1.62, 0.25)	-0.65 (-1.58, 0.28)
	ECV (>28%)	logistic	0.76 (0.24, 2.34)	0.98 (0.87, 1.101)
Echocardiography				
	GLS	Linear	0.31 (-0.76, 1.37)	0.53 (-0.49, 1.56)
	GLS (>-18%)	Logistic	1.34 (0.63, 2.88)	1.09 (0.92, 1.30)

[*] Analyses are adjusted includes adjustments for age, radiotherapy (none versus left-sided or right-sided), cumulative doxorubicin equivalent dosage, trastuzumab treatment, study (PACT vs PACES) and the presence of cardiovascular risk factors (hypertension, hypercholesterolemia, diabetes mellitus, obesity and being a current smoker; none versus 1 or >1).
[†] GLS and ECV measurements could not be completed in 17.1% (31/181) and 11.6% (21/181) of all participants, respectively.

Supplementary Table 4. Physical activity patterns per study group in the Pact-Paces-Heart study.
Presented as median [interquartile range].

	Control N=72	Mod.-to high-int. EX N=80	low-int. EX N=29
Current physical activity (self-reported) [a]			
Moderate-to high-intensity leisure and sport physical activity, min/week[b]	155 [60-360]	150 [60-368]	240 [30-360]
Physical activity in the distant past (face-to-face interview)[c]			
Total MET-hours/week	259 [227-295]	266 [236-298]	246 [227-282]
Moderate-to high-intensity MET-hours/week	86 [56 -126]	94 [63-131]	72 [44-120]

[a] Data on leisure and sport physical activity data were missing in 7/181 (3.9%) participants.
[b] Moderate-to high-intensity activities were defined as activities that corresponded with a metabolic equivalent task (MET) -value of 3 and higher.
[c] Interviews were not conducted in 4/181 (2.2%) due to logistic constraints.

Supplementary table 5. Association between self-reported moderate-to high-intensity leisure and sport physical activity at follow-up and cardiovascular outcomes.

Imaging modality	Parameter	Regression model[a]	Unadjusted estimate (95%CI)	Fully adjusted[b] estimate (95%CI)
Cardiac MRI				
	ECV	Linear	0.11 (-0.04, 0.25)	0.12 (-0.03, 0.27)
	ECV (>28%)	logistic	1.06 (0.91, 1.23)	1.08 (0.92, 1.25)
	Native T1	Linear	-0.20 (-2.66, 2.26)	-0.14 (-2.61, 2.34)
	Native T1 (>1020 ms)	Logistic	0.97 (0.87, 1.08)	0.96 (0.86, 1.06)
	LVEF	Linear	-0.15 (-0.48, 0.18)	-0.11 (-0.45, 0.23)
	LVEF (<50%)	logistic	1.06 (0.94, 1.19)	1.06 (0.93, 1.20)
Echocardiography				
	GLS	Linear	0.14 (-0.01, 0.30)	0.15 (-0.02, 0.31)
	GLS (>-18%)	Logistic	1.08 (0.97, 1.23)	1.08 (0.97, 1.22)
Cardiopulmonary exercise testing				
	VO_2peak	Linear	**0.53 (0.21, 0.85)**	**0.70 (0.42, 0.98)**

[a] All estimates are presented per 100 min/week of reported activity.
[b] Adjusted for age, radiotherapy (none versus left-sided or right-sided), cumulative doxorubicin equivalent dosage, trastuzumab treatment, study (PACT vs PACES) and the presence of cardiovascular risk factors (hypertension, hypercholesterolemia, diabetes mellitus, obesity and being a current smoker; none versus 1 or >1).
